# Association of ultra-processed food patterns with overweight and obesity in the German health interview and examination survey for children and adolescents (KiGGS): a longitudinal study

**DOI:** 10.1186/s12889-025-25181-y

**Published:** 2025-11-17

**Authors:** Mayra Figueiredo Barata, Gert B.M. Mensink, Anja Schienkiewitz, Almut Richter, Renata Bertazzi Levy

**Affiliations:** 1 Robert Koch Institute, Berlin, Germany; 2 Preventive Medicine Department, Medical School, University of Sao Paulo, Sao Paulo, Brazil; 3 Center for Epidemiological Research in Nutrition and Health, University of São Paulo, São Paulo, Brazil; 4 Institute of Biomedical Research of Salamanca (IBSAL), University of Salamanca, Salamanca, Spain

**Keywords:** Ultra-processed food, Child, Adolescent, Overweight, Obesity

## Abstract

**Background:**

Ultra-processed foods (UPF) consumption has been linked to adverse health outcomes, but evidence in children and adolescents from longitudinal studies remains limited. This study aimed to investigate the prospective associations between exposure to an UPF pattern and the incidence of overweight and obesity among German children and adolescents.

**Methods:**

We analyzed 4,762 participants aged 3–17 years from a national cohort with baseline dietary data and anthropometric measures, followed for an average of 11 years. UPF intake was estimated from a Food Frequency Questionnaire using the Nova classification. Logistic regression models assessed associations with incident overweight and obesity, adjusting for age, sex, socioeconomic status, physical activity, and baseline BMI z score.

**Results:**

Each 10% increase in UPF consumption was associated with 12% higher odds of overweight (OR 1.12, 95% CI 1.03–1.23). Compared with the lowest quartile, participants in the highest quartile had 49% greater odds of overweight (OR 1.49, 95% CI 1.05–2.11). For obesity, odds were more than doubled in the third quartile (OR 2.32, 95% CI 1.38–3.88) and 74% higher in the fourth quartile (OR 1.74, 95% CI 1.06–2.84).

**Conclusions:**

Higher exposure to UPF in childhood and adolescence was associated with greater risk of developing overweight and obesity. Strategies to limit UPF exposure may contribute to obesity prevention in this age group and later in life.

**Supplementary Information:**

The online version contains supplementary material available at 10.1186/s12889-025-25181-y.

## Introduction

The prevalence of overweight and obesity among children and adolescents is a major public health concern. In 2022, over 390 million individuals aged 5–19 years were classified as having overweight or obesity, with the prevalence increasing from 8% in 1990 to 20% in 2022 [1]. Obesity alone increased from 2% (31 million) to 8% (160 million) over the same period [1]. Obesity is a complex, multifactorial condition influenced by a dynamic interplay of genetic, environmental and social factors [2–4]. One important contributor to the obesity epidemic, particularly among children and adolescents, is diet [5].

In recent decades, global dietary patterns have shifted significantly, with increased consumption of ultra-processed foods (UPF), which has been closely linked to rising obesity rate [6, 7]. The ultra-processed food as stated by the Nova Food Classification, are industrial formulations that undergo extensive processing, where whole foods are broken down into chemical components, modified and recombined with additives. These products are energy-dense, often nutrient-poor foods, and serve as alternatives to minimally processed foods, processed foods, and freshly prepared meals from whole ingredients [8]. Designed for convenience, these items are typically ready-to-eat, have extended shelf life, and are engineered for hyperpalatability. Their appeal is further enhanced by sophisticated packaging and aggressive marketing strategies, particularly those that target children and adolescents. These combined factors make ultra-processed foods highly competitive in the food market, contributing to their widespread availability across various retail settings [8, 9].

A recent umbrella review of meta-analyses revealed a consistent link between an ultra-processed food pattern and adverse health outcomes, based mainly on studies in adults (cohort, case-control, and cross-sectional designs), with additional evidence from studies in adolescents for mental and respiratory health outcomes (cross-sectional designs). Higher exposures to ultra-processed food are associated with increased risks of overweight and obesity, cardiometabolic disorders, certain cancers and all-cause mortality [10]. A controlled randomized trial demonstrated that an ultra-processed food pattern indeed leads to excess caloric intake and weight gain. Participants consuming ultra-processed foods ad libitum ingested approximately 500 kcal/day more than those on an unprocessed diet did, despite meals being matched for calories, macronutrients, and palatability [11].

The increasing availability and affordability of ultra-processed food have made them especially accessible to younger people (i.e., children and adolescents), who are particularly vulnerable to the effects of poor dietary habits and are currently among the leading consumers in some countries [6, 12–16]. Furthermore, dietary habits established during childhood and adolescence often persist into adulthood, potentially influencing the long-term risk of obesity and other noncommunicable diseases [17–21]. Understanding the complex factors contributing to obesity is essential for developing targeted interventions to mitigate its increasing global prevalence.

Regarding the population of children and adolescents, several studies have explored the relationship between exposure to an ultra-processed food pattern and obesity, yet the results remain somewhat inconsistent [22, 23]. Recently, a review conducted by Petridi et al.^22^ reported generally positive associations between ultra-processed food consumption and overweight/obesity in children and adolescents but suggested that this association presents more robust results in studies in the adult population. Additionally, another systematic review revealed differences in findings, with some studies reporting no clear link between ultra-processed food consumption and obesity parameters in children and adolescents, mainly from cross-sectional studies [23].

These conflicting findings highlight the need for further research to elucidate further the relationship between the exposure to an ultra-processed food pattern and health outcomes in children and adolescents. Given that obesity develops over long periods of time, cross-sectional studies are limited in their ability to assess the relationship between an ultra-processed food pattern and the development of obesity. Therefore, more studies, particularly those with robust longitudinal designs, are essential to strengthen the evidence and improve our understanding of the factors influencing these associations in this age group. In light of this, the aim of this study was to investigate the prospective associations between exposure to an ultra-processed food pattern and the incidence of overweight and obesity among German children and adolescents.

## Methodology

### Study design and sample

The German Health Interview and Examination Survey for Children and Adolescents (KiGGS) was designed to monitor the health status of individuals aged 0–17 years and was conducted by the Robert Koch Institute [24]. KiGGS includes cross-sectional and a longitudinal component. Its longitudinal component, the KiGGS cohort [24], followed a nationally representative sample of 17,640 children and adolescents who participated in the KiGGS baseline study (2003–2006) into young adulthood through subsequent follow-ups. The baseline study was conducted as an examination and in-person interview survey. The first follow-up, KiGGS Wave 1 (2009–2012), was conducted as a telephone survey, whereas the second follow-up, KiGGS Wave 2 (2014–2017), reintroduced in-person interviews and physical examinations. The design and methodology of KiGGS have been described in detail elsewhere [24]. The study protocol was approved by the Federal Office for Data Protection. Ethical approval was obtained from the ethics committee of the Charité-Universitätsmedizin Berlin for KiGGS baseline [24] and from The Hanover Medical School’s ethics committee for KiGGS Wave 2 [25]. Written informed consent was obtained from all participants aged 14 years or older and from the parents or legal guardians of younger participants before any interviews or examinations were conducted [24, 25].

All participants from the baseline study who consented to further contact were invited to participate in KiGGS Wave 2, regardless of their participation in KiGGS Wave [1] However, only those who indicated a willingness to be interviewed again, who could be located, and who still lived at the original sample point were invited to both the physical examinations and the interviews in KiGGS Wave [2] In total, 6465 individuals out of the baseline sample completed the physical examinations and the interviews in KiGGS Wave 2 [25].

### Data collection

The KiGGS cohort involved comprehensive health interviews and physical examinations, capturing a broad spectrum of information on physical and mental health, health behaviors, psychosocial factors, and sociodemographic characteristics. For all the participants, parents or legal guardians completed self-administered questionnaires covering their child’s health, health behaviors, and sociodemographic background. Adolescents aged 11 to 17 years provided additional information via self-administered questionnaires. Physical examinations were conducted by trained staff, who collected standardized anthropometric measurements, including body height and weight [24, 26].

#### Assessment of exposure to ultra-processed food pattern

Food consumption was assessed at baseline via a validated 45-item food frequency questionnaire (FFQ) [27]. For each food item, participants reported their consumption frequency over the past four weeks using 10 response options (never, once per month, 2–3 times per month, 1–2 times per week, 3–4 times per week, 5–6 times per week, once per day, 2–3 times per day, 4–5 times per day, and more than 5 times per day) and the portion size consumed. Portion sizes were illustrated via household measures (e.g., cups, tablespoons) and pictures [27].

The food consumption frequencies were converted into the number of consumption occasions over a four-week period (28 days). Portion sizes were converted into grams. The estimated daily gram intake for each food item was calculated by multiplying the consumption frequency by the portion size and dividing the result by 28 [27, 28]. For example, if a participant reported consuming one portion of cooked potatoes (175 g) one or two times per week (corresponding to a consumption frequency of 6), the estimated daily intake of cooked potatoes would be calculated as (175 × 6)/28, resulting in approximately 37.5 g/day.

Furthermore, the total energy intake was calculated on the basis of the food item intake and the mean energy contents of the food items. The mean energy content of each food item was derived from weighted estimates of consumption amounts of foods within these food groups on the basis of consumption data from a more comprehensive consumption survey (EsKiMo) (e.g., combining the energy content of different types of bread into the total bread quantity) [29, 30]. The Nova classification system was used to categorize the FFQ items into two groups based on the degree of industrial processing: (i) unprocessed or minimally processed foods and beverages, culinary ingredients and processed foods and beverages and (ii) ultra-processed foods and beverages [8]. The exposure to the ultra-processed food pattern was estimated by the dietary contribution of ultra-processed food to total energy intake, which was analyzed both as quartiles and as a continuous variable, expressed per 10% increase in consumption.

To address missing data in the FFQ, an imputation procedure was conducted using criteria partly determined by the authors and partly based on previously published studies using a similar method [28, 29]. The process was applied individually to each food or beverage item, and the frequency variables were used as the primary reference. Participants with more than 30% missing frequency responses (i.e., more than 14 missing out on 45 items) were excluded from the analysis. In the final analytic sample, 13.9% of the participants had at least one missing frequency value. For each item, sex- and age-specific mean values were used to impute missing responses, according to the following rules: (a) For missing frequency only: if the frequency of consumption was missing, but the reported portion size was available, the frequency was set to the age- and sex-specific average frequency for that item; (b) For missing portion size only: if the portion size was missing, but the frequency was available, the portion size was set to the age- and sex-specific average portion size; (c) For both values missing: when both frequency and portion size were missing for a given item, they were set to non-consumption.

#### Anthropometric assessment

Anthropometric measurements were conducted by trained staff at baseline and in Wave 2. Height was measured to the nearest 0.1 cm using a portable Harpenden stadiometer (Holtain Ltd., Crymych, UK) while the participants were barefoot. Weight was measured to the nearest 0.1 kg using a calibrated electronic scale (SECA, Birmingham, UK), with participants wearing only underwear and no shoes. Body mass index (BMI) was calculated as weight (kg) divided by height squared (m²) [24].

In Germany, overweight and obesity among children and adolescents are defined based on the Kromeyer-Hauschild percentile curves [31, 32]. Accordingly, at baseline and follow-up, children and adolescents were classified as overweight if their BMI fell between the 90th and 97th percentiles of the reference population, and as having obesity if their BMI exceeded the 97th percentile, for a specific age and sex. After an average follow-up of 11 years, several participants had reached adulthood. For these individuals, overweight was defined as a BMI between 25.0 and 29.9 kg/m² and obesity as a BMI ≥ 30.0 kg/m², following the WHO classification [33]. The incidence of overweight (including obesity) at follow-up was assessed among participants without excess body weight (i.e., with overweight or obesity) at baseline. The incidence of obesity was analyzed separately among those who did not have obesity at baseline.

#### Assessment of other variables

Health behaviors and sociodemographic background at baseline included age, sex, socioeconomic status, and physical activity. Socioeconomic status was determined using a multidimensional index based on parental information regarding educational attainment, occupational status, and household net income. The index was calculated as the sum of point scores assigned to equally weighted subscales of the three dimensions. SES index values ranged from 3.0 to 21.0 and could be used as a continuous variable or categorized into socioeconomic groups. For this study, following the previous survey categorization, the index was classified into lower (first quintile of the SES distribution), middle (second to fourth quintiles), and higher socioeconomic status groups (fifth quintile) [34]. The physical activity variable was derived from three age-specific questions included in the study questionnaire. For children aged 3–10 years, the frequency of activity was assessed within and outside a sports club, whereas for adolescents aged 11–17 years, leisure-time physical activity frequency was evaluated. The responses were harmonized into a single variable with three categories: inactive (no activity reported for both contexts in children or leisure-time physical activity in adolescents), partially active (low to moderate frequency, such as rarely or 1–2 times per week), and active (high frequency, such as three or more times per week).

Multiple imputations were used to impute missing covariate data under the assumption of missing data at random, and sex and age were used as predictor variables. This included socioeconomic status (0.56% from the final analytical sample) and physical activity (1.73% from the final analytical sample).

#### Data analysis

**Fig. 1:**
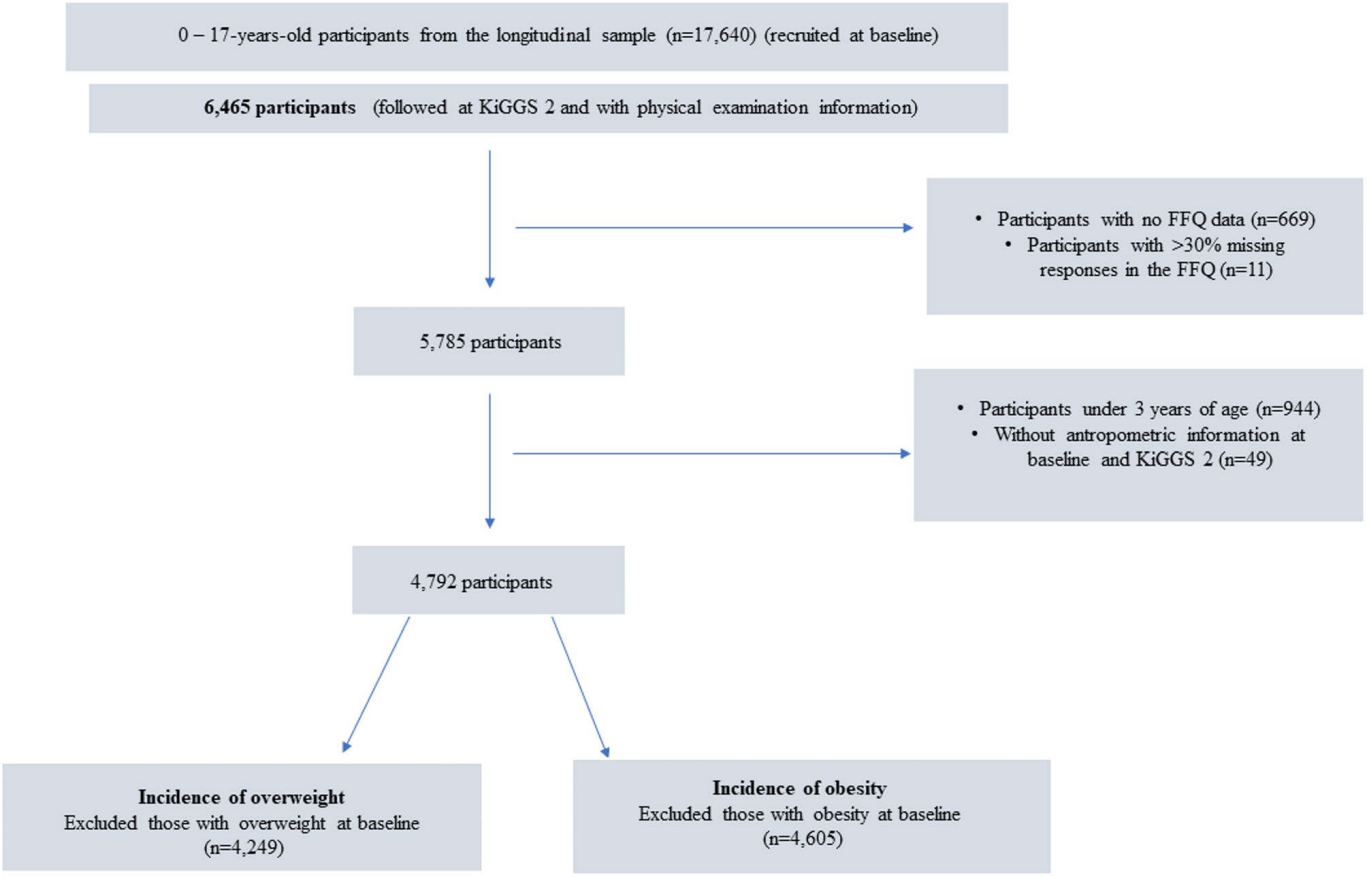
Flowchart of the participants in the present study

The analyses were based on data from 4,762 participants aged 3–17 years with valid food intake information at baseline and anthropometric measurements at both baseline and Wave 2. For the overweight analyses, participants classified as having excess body weight (i.e., with overweight or obesity) at baseline were excluded, resulting in an analytical sample of 4,249 participants. Similarly, for the obesity analyses, those with obesity at baseline were excluded, resulting in a sample of 4,605 participants (see Fig. 1).

The baseline characteristics of the study population were described according to quartiles of exposure to the ultra-processed food pattern. For descriptive analysis, continuous variables were summarized as means and standard errors (SEs), whereas categorical variables were presented as percentages. Trends for those characteristics across ultra-processed food consumption quartiles were assessed using linear regression models.

Furthermore, logistic regression models were applied to prospectively assess the exposure to dietary patterns divided into quartiles of ultra-processed food consumption (based on the analytic sample and as reference category: participants in the first quartile) and the incidence of overweight and obesity. All the models were adjusted for age, sex, socioeconomic status, physical activity, and the baseline BMI z score (Model 1). Additional analyses were performed with further adjustment for total energy intake (Model 2). Subsequently, linear trends for overweight and obesity were assessed by coding quartiles as continuous predictors via logistic regression (p-trend).

Further, the assumption of linearity between exposure to the ultra-processed food pattern (10% increase in dietary energy intake of ultra-processed food) and both outcomes was evaluated using restricted cubic splines functions [35]. The statistical significance of the linear and nonlinear terms was evaluated using Wald tests. No statistically significant violation from the linearity assumption was observed (Wald tests for nonlinear terms *p* > 0.05) (Additional file 1). A weighting factor was applied in all the statistical analyses to account for potential biases due to selective participation over time and to adjust for the clustered sampling design; survey commands were used for these adjustments. This process generated a weighted sample reflecting the KiGGS baseline population [36]. All analyses were conducted using STATA/SE version 17, and *p* < 0.05 was considered statistically significant.

## Results

Table 1 describes the dietary intake and BMI categories of the study population according to quartile of exposure to the ultra-processed food pattern at baseline. The estimated mean total energy intake was 2,126 kcal/day (SE: 29.8). Ultra-processed foods contributed a mean of 54.7% of the total daily energy intake. Regarding sociodemographic characteristics, compared with participants in the first quartile (lower ultra-processed food intake), those in the highest quartile were more often boys (55.6% vs. 43.7%), had a higher proportion of low socioeconomic status (27.1% vs. 9.8%), and were more frequently classified as inactive (32.1% vs. 28.0%) (Additional File 2).

**Table 1:** Baseline dietary intake and BMI categories by ultra-processed food quartiles. KiGGS, 2003–2006

			Quartiles of ultra-processed food pattern exposure (% dietary energy intake of UPF)								
	All participants		Q1		Q2		Q3		Q4		*p*-value ^b^
Characteristics			(16.79–46.16) ^**a**^		(46.40–55.36) ^**a**^		(55.52–65.05) ^**a**^		(65.24–89.68) ^**a**^		
Total energy intake, kcal/day (mean, SE)	2,112 (29.8)		2,065 (40.9)		1,928 (50.2)		2,024 (49.6)		2,430 (68.7)		< 0.001
Mean ultra-processed food consumption (%, 95% CI)											
relative to total daily energy intake	55.3	54.7–55.9	37.7	37.1–38.3	50.9	50.7–51.1	60.0	59.8–60.2	72.5	72.0–73.0	< 0.001
Weight status (%, 95% CI) ^c^											
Underweight (< P10)	7.21	6.25–8.29	5.88	4.54–7.58	7.02	5.31–9.22	8.71	6.76–11.2	7.23	5.30–9.78	0.271
Normal weight	77.6	75.9–79.3	78.5	75.0–81.7	76.5	73.1–79.6	76.4	72.8–79.7	79.2	74.9–82.8	
Overweight (*P* > 90 - P97)	8.99	7.86–10.3	10.0	7.54–13.1	9.19	7.21–11.7	9.10	6.90–11.9	7.68	5.63–10.4	
Obesity (*P* > 97)	6.16	5.06–7.48	5.62	3.82–8.22	7.29	5.22–10.1	5.79	4.11–8.11	5.93	3.82–9.10	

a: Range of the contribution of ultra-processed foods on total energy intake in each quartile (weighted)

b: p-value: The values in this column refer to the test for linear trend

c: Weight status was classified according to the Kromeyer-Hauschild percentiles for children and adolescents: underweight (< P10), normal weight (P10–P90), overweight (P90–P97), and obesity (> P97)

The foods and beverages classified as ultra-processed food are presented in Additional File 3. Breads (both white and brown), yogurt and soft drinks were the major contributors to total energy intake, each accounting for more than 4.5%, with breads reaching a total contribution of 12.8%. Other items contributing more than 3.5% included chocolate, breakfast cereals, and sausages/hams.

**Table 2:** Associations between ultra-processed food pattern and incident overweight/obesity after a mean 11-year follow-up. KiGGS, 2003–2017

	Quartiles of ultra-processed food pattern exposure (% dietary energy intake of UPF)									
	Q1	Q2		Q3		Q4		*p* for trend	For each 10% point increase	
Incident overweight ^+, a^										
* n for cases/total n*	189/1,233	217/1,163		195/1,019		189/835			790/4,249	
Incidence (%) ^c^	18.5	24.3		24.2		26.1			23.3	
Crude Model (OR, 95% CI)	1.00 (Ref.)	1.27	(0.91–1.78)	1.55	(1.12–2.14)	1.53	(1.12–2.09)	0.005	1.12	(1.03–1.21)
Model 1 (OR, 95% CI)	1.00 (Ref.)	1.25	(0.86–1.80)	1.52	(1.07–2.15)	1.49	(1.05–2.11)	0.019	1.12	(1.03–1.23)
Model 2 (OR, 95% CI)	1.00 (Ref.)	1.27	(0.87–1.84)	1.53	(1.08–2.17)	1.49	(1.05–2.12)	0.017	1.12	(1.03–1.22)
Incident obesity ^§, b^										
* n for cases/total n*	72/1,328	81/1,260		70/1,101		66/916			289/4,605	
Incidence (%)^c^	6.33	7.14		8.78		7.91			7.54	
Crude Model (OR, 95% CI)	1.00 (Ref.)	1.75	(1.06–2.90)	2.13	(1.30–3.51)	1.57	(1.00–2.47)	0.065	1.05	(0.95–1.16)
Model 1 (OR, 95% CI)	1.00 (Ref.)	1.74	(1.00–3.02)	2.32	(1.38–3.88)	1.74	(1.06–2.84)	0.002	1.10	(0.98–1.23)
Model 2 (OR, 95% CI)	1.00 (Ref.)	1.74	(1.00–3.04)	2.32	(1.38–3.90)	1.74	(1.06–2.84)	0.019	1.10	(0.98–1.24)

*UPF* ultra-processed foods, *OR* odds ratio, *CI* Confidence Interval

a: Overweight (including obesity) was defined as follows: for adolescents, BMI-for-age percentile > 90th based on Kromeyer-Hauschild references, for adults, BMI ≥ 25 kg/m²

b: Obesity was defined as follows: for adolescents, BMI-for-age percentile > 97th based on Kromeyer-Hauschild references, for adults, BMI > 30 kg/m

c: Weighted

+ Among those without excess of weight at baseline

§ Among those without obesity at baseline

Model 1: adjusted by sex, age, socioeconomic status, physical activity and baseline BMI z-score

Model 2: Model 1 + total energy intake

The associations between the dietary contribution of ultra-processed foods and the incidence of overweight and obesity are shown in Table 2. In the continuous model presented for the overweight analysis, associations were analyzed for a 10% increase in total energy intake from ultra-processed food. In the crude model, a 10% increase in ultra-processed food consumption was associated with increased odds of becoming overweight at follow-up (OR: 1.12, 95% CI: 1.03–1.21). After adjusting for potential confounders (Model 1), the association remained significant and essentially the same (OR: 1.12, 95% CI: 1.03–1.23). When ultra-processed food consumption was analyzed in quartiles, individuals with higher exposure to UPF intake (Q4) had 49% higher odds of becoming overweight (OR: 1.49, 95% CI: 1.05–2.11) than those with the lowest exposure to ultra-processed food intake (Q1) (Model 1). A significant dose‒response trend was observed across quartiles (*p* for linear trend across quartiles < 0.05).

With respect to incident obesity, a uniformly graded increase across quartiles of exposure was not observed. However, individuals in the third and fourth quartiles of exposure to an ultra-processed food pattern had significantly greater odds of obesity at follow-up, with ORs of 2.32 (95% CI: 1.38–3.88) and 1.74 (95% CI: 1.06–2.84), respectively.

The spline plot for overweight remained relatively uniform across the exposure range. Conversely, for obesity, the spline suggested a potential shift in the association at higher levels of ultra-processed food intake, where the odds appeared to slightly decrease. However, statistical tests (Wald tests for nonlinear terms) did not detect a significant deviation from linearity (*P* > 0.05), and the confidence intervals broadened at the extremes. Therefore, these visual patterns should be interpreted cautiously (Additional File 1).

## Discussion

Findings from this prospective study of children and adolescents in Germany indicate that higher exposure to ultra-processed food intake was associated with an increased likelihood of developing overweight and obesity over an average follow-up period of 11 years. A 10% increase in ultra-processed food contribution to energy intake was linked to a 12% greater risk of developing overweight, with a clear dose‒response relationship. Higher ultra-processed food consumption was also associated with increased risk of obesity.

The findings from this study align with studies that have investigated the association between exposure to ultra-processed food patterns and obesity-related outcomes in children and adolescents across different populations. In China, a study of children and adolescents initially aged 6 − 18 years between 1997 and 2011 (*n* = 3,437) revealed that higher ultra-processed food intake was associated with a twofold increased risk of overweight and obesity [37]. Findings from a prospective birth cohort in Portugal reported a positive association between higher ultra-processed dietary exposure at age 4 and an elevated BMI z score at age 10, suggesting that early exposure to ultra-processed food may contribute to increased obesity later in childhood. The study followed 1,175 children with assessments at ages 4, 7, and 10 years [38]. Chang et al.^39^ analyzed data from 9,025 children followed from ages 7 to 24 years over a median period of 10.2 years in England (1998–2017). The study reported that higher ultra-processed food exposure was associated with an additional increase of 0.06 kg/m² in BMI per year, highlighting the long-term impact of UPF intake on BMI trajectories [39].

However, not all studies have reported consistent results. A longitudinal study conducted in Brazil among adolescents aged 15 − 17 years at baseline observed an inverse association between ultra-processed food consumption and both BMI and body fat percentage over a 3-year follow-up [40]. The study included 1,035 participants in the first year, 787 in the second year, and 585 in the third year. Similarly, findings from the Kiel Obesity Prevention Study (KOPS), conducted in Kiel, Germany, with 182 participants followed into young adulthood (aged 23–31 years), indicated that an ultra-processed food pattern was inversely associated with BMI and the fat mass index (FMI) over a follow-up period of 13.3 years [17]. These results could be explained by higher levels of physical activity observed among adolescents in the highest quartile of ultra-processed food consumption [40], or they may reflect selection bias, as participants in the follow-up of the KOPS study had lower BMIs and higher socioeconomic statuses than did the baseline population [17]. These factors may have limited the ability to observe long-term adverse health outcomes associated with exposure to ultra-processed food patterns.

Beyond BMI, other studies have explored the effects of ultra-processed food contributions to total energy intake on body composition. Costa et al.^41^ reported that ultra-processed food contribution was associated with increased waist circumference from preschool (age 4) to school age (age 8) in Sao Leopoldo, Brazil, although no association was observed with changes in BMI. This study followed 500 children from birth, with 354 assessed at age 4 and 315 at age 8 [41]. Furthermore, data from the Pelotas-Brazil Birth Cohort demonstrated that daily UPF consumption between the ages of 6 and 11 was positively associated with a 0.14 kg/m² increase in the fat mass index (FMI). At ages 6 and 11, the study included 3,128 and 3,454 participants, respectively [41].

Several mechanisms related to ultra-processed food can help explain the associations found between their consumption and increased risk for overweight/obesity. First, their nutritional profile and the replacement of low caloric, nutritious and minimally processed foods from the diet may contribute to weight gain [42, 43]. A meta-analysis indicated that dietary patterns based on ultra-processed foods were associated with higher total energy intake and a decline in overall dietary nutritional quality. Higher ultra-processed food intake was linked to higher levels of free sugars and total and saturated fats and lower intakes of fiber, protein, key minerals, and several vitamins [44]. Moreover, the higher energy density and sensory characteristics of ultra-processed food (i.e., softer, less fibrous textures that are easy to chew) could also enable greater energy intake in a shorter amount of time [42, 45]. Additionally, convenience, omnipresence, affordability, and large portion sizes may promote poor dietary habits, snacking, and overeating, potentially contributing to increased energy intake [45, 46]. These combined unfavorable ultra-processed food characteristics are intensified by persuasive and sophisticated marketing, especially among the most vulnerable consumers, such as children [47]. All these characteristics may explain how ultra-processed food lead to increased energy intake. Nevertheless, the presented results were adjusted for total energy intake, and the associations continued to be significant, which may suggest that additional mechanisms could play a role.

Second, the industrial processing techniques used in ultra-processed food production disrupt the food matrix, significantly altering the raw materials. For example, the removal of water is a common process that impairs the brain’s perception of the ingested nutritional content, thereby affecting satiety-regulating mechanisms. Furthermore, the low water content of many ultra-processed foods contributes to a reduction in total water intake, as these products frequently replace meals based on minimally processed foods [44, 48]. Additionally, changes in the physical structure of the food matrix can have consequences for the nutrient bioaccessibility and absorption kinetics, altering gut microbiota composition, metabolism, and growth [42, 49, 50]. Experimental studies suggest that the large share of acellular nutrients in ultra-processed foods leads to a high nutrient availability in the small intestine, which, in turn, promotes an inflammatory gut microbiota associated with obesity and other inflammatory diseases [49–51].

Third, cosmetic additives in ultra-processed foods are likely to play an important role in the biological mechanism promoting obesity. Emulsifiers such as carboxymethylcellulose and polysorbate-80 have been shown to disrupt the intestinal mucus barrier in mice, leading to low-grade inflammation and metabolic syndrome, a phenotype linked to weight gain [52]. Similarly, monosodium glutamate, a common flavor enhancer, may have endocrine-disrupting effects that contribute to obesity [53]. The increased consumption of food additives has also been linked to alterations in the composition of the gut microbiota, potentially leading to metabolic dysfunction [54–56]. For example, soft drink consumption, which involves the consumption of multiple additives, has been associated with lower levels of *Akkermansia muciniphila*, a bacterium thought to protect against obesity and type 2 diabetes [57, 58]. Additionally, snack and junk foods, often containing a wide range of additives, have been linked to an increased presence of *Escherichia coli* and a reduced abundance of beneficial bacteria, such as lactobacilli and butyrate-producing *Firmicutes* species, changes believed to promote gut inflammation and metabolic disturbances [57].

Finally, ultra-processed foods contain xenobiotic substances due to the plastic packaging in which they are typically wrapped. Higher concentrations of phthalates and bisphenol have been linked to increased UPF consumption [59], and these substances have been associated with obesity [60, 61].

In this study, food consumption was assessed between 2003 and 2006, while the outcome was analyzed between 2014 and 2017, after a mean follow-up period of 11 years. Dietary habits are known to change substantially during adolescence and early adulthood [23]. Although the odds of both overweight and obesity observed in the present study are substantial, this time gap between exposure and outcome in such an age group was not captured and may have introduced some regression dilution bias. Some participants may have reduced their intake, which could have added variability and further diluted the associations. Conversely, participants may have increased their ultra-processed food consumption, given the continued rise in availability and consumption of ultra-processed foods and their rapid global expansion. In that case, the magnitude of the association may be even greater in today’s context. Sales of ultra-processed foods and beverages have increased across nearly all regions of the world. Between 2006 and 2019, all regions experienced growth in the per capita sales volume of ultra-processed foods [6]. In 2019, the total per capita sales volume of ultra-processed foods in high-income countries was 3.4 and 11.3 times greater than that in upper-middle- and low-income countries, reaching 109.3, 32.3, and 9.7 kg per capita sales per year, respectively. Among high-income countries, the United States (246.0 kg per capita per year) and Germany (171.4 kg per capita per year) recorded the highest per capita sales volumes of ultra-processed foods and beverages [6]. Taken together, these findings highlight that our study, while based on earlier dietary assessments, still captures the important role of early exposure to ultra-processed foods on long-term weight outcomes, which was our main goal. The rising global consumption of these products only reinforces their significance as a key determinant of nutrition and human health. From a public health perspective, ultra-processed foods require increased attention, including continuous monitoring and the implementation of strategies aimed at mitigating their potential negative long-term effects on population health.

We found notable contrasts between overweight and obesity outcomes that require careful consideration. Associations between ultra-processed food consumption and overweight remained robust, even after adjusting for total energy intake, whereas the corresponding associations with obesity were less consistent and not clearly linear. Several factors may help explain this difference. First, our cohort was relatively healthy at baseline, with low obesity prevalence and a limited number of incident cases over follow-up. This reduced statistical power and led to wider confidence intervals, reflecting that obesity estimates were more variable than those for overweight. Second, overweight may act as an earlier and more sensitive marker of excess weight gain, which could explain the clearer and more linear associations observed in our study. In contrast, obesity may require longer exposure to ultra-processed foods or be influenced by additional determinants, such as genetic predisposition or metabolic factors [62], which may help explain why the associations we observed were less linear. Third, methodological aspects and behavioral patterns may have also contributed. In the quartile analyses, for example, individuals in the second quartile reported lower mean energy intake than those in the first quartile, and the obesity results did not follow a clear dose–response gradient, with larger odds ratios in the third quartile than in the fourth. These counterintuitive findings could partly reflect sampling variability, given the relatively small number of incident obesity cases in some quartiles. Indeed, we had already observed unexpected baseline differences in weight status: a higher prevalence of underweight in the third and fourth quartiles; higher prevalence of overweight in the first and second quartile; and the highest prevalence of obesity in the second quartile. Additionally, reporting bias in dietary intake, such as underreporting of ultra-processed foods, and behavioral compensatory patterns could have affected the apparent trends across quartiles. Future research with larger samples and repeated dietary assessments will be important to disentangle these patterns and clarify whether obesity-specific mechanisms are at play.

Regarding our choice of analysis, we acknowledge that other longitudinal approaches could have been considered, such as generalized estimating equations or mixed-effects models, which are commonly used in longitudinal studies [63, 64]. However, given the characteristics of the survey, with baseline and a single follow-up point, the use of logistic regression allowed us to test our hypothesis and present the results in a scientifically robust manner. This approach, or similar, has been used in previous studies with comparable longitudinal designs [65, 66].

In the spline analyses, a generally consistent pattern across the exposure range was observed for overweight, and statistical tests did not indicate meaningful deviations from linearity. For obesity, a potential change in the odds at higher levels of ultra-processed food intake was suggested visually. However, Wald tests did not support a significant non-linear association. These findings suggest that the observed changes in odds at the highest levels of ultra-processed food intake may reflect data sparsity or variability in this exposure range, rather than a true deviation from overall linear association.

Our analyses were based on a prospective study design and used data from a nationally representative population sample of children and adolescents in Germany to analyze the associations between an ultra-processed food pattern and overweight and obesity. Nevertheless, potential limitations should be considered. First, dietary intake was assessed using a food frequency questionnaire (FFQ), which, although widely used in epidemiological studies, has inherent limitations. Typically, FFQs are susceptible to measurement errors, including recall bias and misreporting, which can lead to under- or overestimation of food intake and total energy intake. The FFQ used in our study provides a broad estimation of dietary intake and it was not originally designed to specifically evaluate the degree of food processing, which may lead to some degree of misclassification. For example, certain food groups such as juices and breads are heterogeneous and may contain both minimally processed and ultra-processed varieties. This limitation could result in an over- or underestimation of the ultra-processed food consumption. Nevertheless, previous research has consistently demonstrated the relevance of assessing ultra-processed food consumption as a group and through relative measures, emphasizing that it is not only individual ingredients or nutrients, but the combination of characteristics of ultra-processed foods that negatively impacts health [67, 68]. To address potential biases, we applied strict, systematic procedures for the classification of FFQ items, following established methodological recommendations [67, 69, 70] and expressed ultra-processed foods intake as a percentage of total energy intake. Importantly, the application of Nova to FFQ data, despite these limitations, is widely used in epidemiological studies investigating UPF consumption [65, 69, 70], thus supporting the comparability of our findings with the existing literature. Future dietary and health surveys could benefit from instruments specifically designed to capture food processing levels, such as the NovaFFQ [71] or the NFQQ (adapted to the Italian adult population) [72], which may provide more precise measurements of ultra-processed food intake while accounting for national and cultural dietary characteristics.

Furthermore, unmeasured factors such as socioeconomic changes, family eating behaviors, screen time, and sleep patterns might influence both ultra-processed food consumption and weight outcomes. For example, if children or adolescents with higher ultra-processed food intake also tend to have shorter sleep duration or higher screen time, both of which increase obesity risk, then the observed associations could be overestimated. Another important aspect is the potential role of sexual maturation, which was not accounted for in the analyses. Although puberty affects nearly all children and adolescents, variations in its timing and pace can influence body composition, metabolism, and eating behaviors, and its impact on weight status and dietary patterns may still differ by individual maturation stage, potentially confounding the relationship between ultra-processed food consumption and obesity. However, in this longitudinal design with a relatively large period between exposure and outcome, it is very unlikely that puberty has a substantial impact on the results. More broadly, the direction of potential bias is not always straightforward, as some of these unmeasured factors could amplify, while others may attenuate, the true association between ultra-processed food intake and weight gain. While this limitation is common to most observational studies [65, 73], adjustment for key determinants in our models likely reduced, but did not eliminate, the risk of residual confounding.

Moreover, the reduction in sample size from 17,460 participants at baseline to 6,465 (with completed physical examinations) in Wave 2 indicated considerable loss to follow-up. To mitigate potential biases related to systematic dropout, we applied the survey weighting factor, which was designed to improve sample representativeness. Finally, it is important to acknowledge that dietary habits can change over time, especially during different stages of childhood and adolescence [23]. This variability can introduce challenges in accurately assessing the long-term effects of ultra-processed foods consumption on the basis of only one survey date at baseline. However, the study goal was to analyze the association between exposure to the ultra-processed food pattern in the early stages (at baseline) and the incidence of overweight/obesity after an average of 11 years of follow-up.

Policy measures aimed at increasing the consumption of unprocessed and minimally processed foods and restricting access to and advertising ultra-processed foods, thereby reducing their consumption, may be particularly beneficial for children and adolescents. Some countries have already implemented successful strategies in this regard. In Brazil, the National School Feeding Program provides free meals to all students in public schools (primary, middle, and high schools). The program limits spending on processed and ultra-processed foods to a maximum of 20% of the total budget; prohibits ultra-processed foods for children up to three years of age; and restricts the offering of soft drinks, chocolate, candies, cookies, cakes with toppings or fillings for all students [74]. In Chile, front-of-package warning labels have been a key regulatory strategy. These labels are placed on prepackaged foods that are high in energy or nutrients linked to noncommunicable diseases. Products with such labels are prohibited from using promotional strategies, such as cartoon characters, on their packaging [75]. Additionally, several countries have introduced restrictions on advertising unhealthy food products during children’s programming or during shows with large child and adolescent audiences [76–78]. In Portugal, advertising for unhealthy food products on websites, social networks, and mobile applications directed at children under sixteen years of age is forbidden [79].

At the European Union level, recent policy initiatives, such as the Farm to Fork Strategy, have committed to harmonized front-of-package nutrition labelling, nutrient-profiling criteria to restrict nutrition and health claims, and strengthened food information rules to support healthier food environments [80]. Implementation across Member States, however, remains heterogeneous, particularly in school food and labelling policies [81–83]. Similarly, although several front-of-package labelling schemes (e.g., Nutri-Score, traffic-light systems) have been adopted in Europe, their uptake is uneven and such interpretative systems, which are most effective for vulnerable groups, are not consistently applied [81, 84]. Besides that, marketing restrictions on unhealthy foods, especially to children, also remain limited, despite repeated calls for stronger regulation [85].

Specifically, in Germany, national guidance and school meal standards exist, but many measures rely on voluntary implementation and industry self-regulation, which evaluations suggest may be insufficient to substantially reduce children’s exposure to ultra-processed foods and related marketing [81, 83]. Given these current gaps on food and nutrition policies in the EU and Germany, our findings highlight the urgency for more consistent, binding measures of food labelling, marketing and school-food regulations, at both the EU and within Germany, to better protect children and adolescents in Germany and contribute to healthier food environments overall.

## Conclusions

This study reinforces evidence on the association between exposure to an ultra-processed food pattern and the incidence of overweight and obesity in children and adolescents, contributing to the growing literature on the effects of ultra-processed food consumption on this age group. Given that dietary habits and their health consequences tend to track into adulthood, early-life interventions are essential for altering these trajectories. Strengthening policies that regulate access, marketing, and consumption of ultra-processed foods may help prevent overweight and obesity in childhood and later in life, supporting long-term public health efforts.

## Supplementary Information

Additional File 1. Spline plots for the associations between ultra-processed food consumption and odds of overweight and obesity. Spline plots for the associations between ultra-processed food consumption and odds of overweight and obesity. Spline plots for the associations between exposure to the ultra-processed food pattern (contribution of ultra-processed foods to total energy intake – % UPF) and odds ratios of overweight and obesity. KiGGS (2003 – 2017). The knots correspond to the 20th, 40th, 60th, and 80th centiles for contributions of 42.38%, 50.16%, 56.92%, and 65.20%, respectively, of ultra-processed foods to total energy intake in the non-overweight sample at baseline (n=4,249) and to 42.36%, 50.07%, 56.84%, and 65.15%, respectively, in the nonobese sample at baseline (n=4,605). Overweight analyses: Wald test for the linear term, P=0.195; Wald test for all nonlinear terms =0.516. Obesity analyses: Wald test for linear term P=0.501; Wald test for all nonlinear terms P=0.066. While plots suggest relatively uniform associations for overweight and a potential non-linear pattern for obesity at higher exposures, Wald tests did not confirm evidence of non-linearity. 

Additional file 2. Table showing baseline sociodemographic and lifestyle characteristics by UPF quartiles.

Additional file 3. Table showing contribution of food and beverages to total energy intake.

## Data Availability

The dataset from the KiGGS Baseline study is available to interested researchers as de facto anonymized data for scientific secondary analysis upon application. Access to longitudinal data from subsequent waves is possible by submitting an informal request along with a description of the proposed project to the ‘Health Monitoring’ Research Data Center at the Robert Koch Institute, Berlin, Germany (e-mail: fdz@rki.de).
