# Supplementary figures and images for: Association of ultra-processed food patterns with overweight and obesity in the German health interview and examination survey for children and adolescents (KiGGS): a longitudinal study
Source: BMC Public Health. 2025 Nov 17;25:3961. doi: 10.1186/s12889-025-25181-y (PMC12621368; doi:10.1186/s12889-025-25181-y)

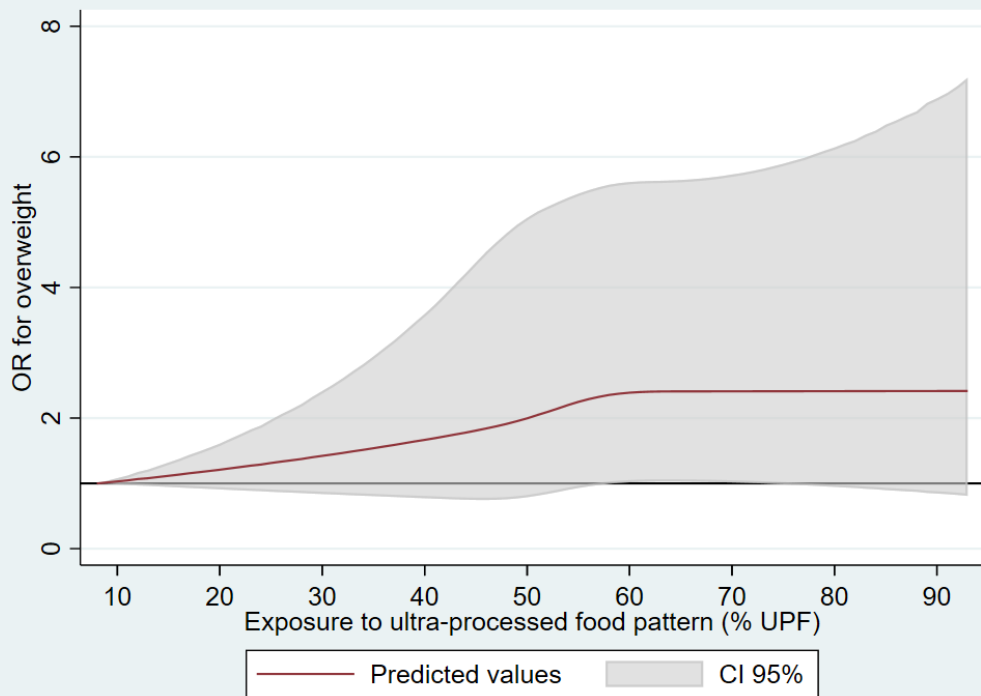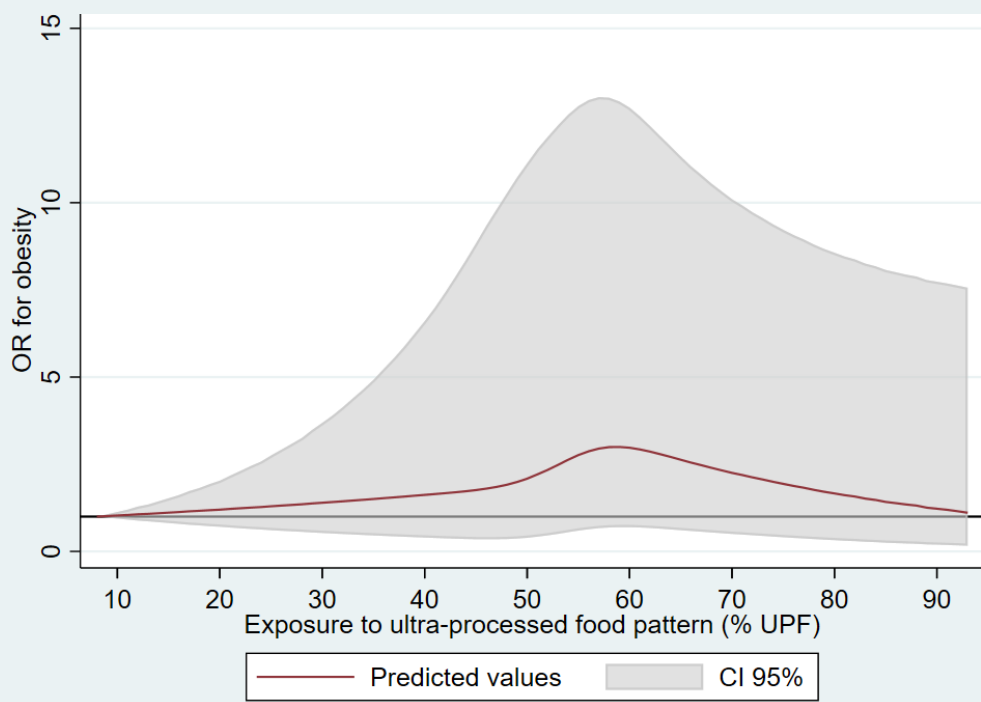

Supplement: Supplementary file 2 — Additional file 2. Table showing baseline sociodemographic and lifestyle characteristics by UPF quartiles. [file 12889_2025_25181_MOESM2_ESM.pdf]
